# Rheoreversible hydrogels in paper restoration processes: a versatile tool

**DOI:** 10.1186/1752-153X-8-10

**Published:** 2014-02-10

**Authors:** Claudia Mazzuca, Laura Micheli, Federico Marini, Marta Bevilacqua, Gianfranco Bocchinfuso, Giuseppe Palleschi, Antonio Palleschi

**Affiliations:** 1 Dipartimento di Scienze e Tecnologie Chimiche, Università di Roma “Tor Vergata”, Via della Ricerca Scientifica snc, Rome 00133, Italy; 2 Dipartimento di Chimica, Università di Roma “Sapienza”, P.le Aldo Moro 2, Rome 00185, Italy

**Keywords:** Rheoreversible hydrogel, Cleaning treatment, Paper artworks, FTIR, HPLC, PCA analysis

## Abstract

**Background:**

Paper based artworks are probably ones of the most difficult materials to restore, because of their complexity and fragile structure. Cleaning of paper artifacts, one of the process commonly carried out during restoration, usually involves the use of solvents (organic or not), that may cause several troubles, like swelling and dissolution of some components, and may also be harmful to the users.

**Results:**

Innovative procedure for cleaning paper artworks is reported in this paper. It is based on the use of rheoreversible, biocompatible hydrogels containing poly(ethylene oxide) or poly(ethylene oxide)-poly(propylene oxide)-poly(ethylene oxide) and α-cyclodextrin. We have studied two types of polymer with different hydrophobic properties in order to obtain two different hydrogels with slightly different cleaning capabilities. Our overall strategy has been to develop innovative systems based on these hydrogels so as to better confront the problems that a restorer faces during the cleaning of paper samples. Rheoreversible hydrogels are intriguing materials because their application and removal is not invasive and does not require a liquid treatment that could induce damage to the paper.

**Conclusions:**

These hydrogels have been applied in the cleaning of both new and aged paper samples and their cleaning efficiency has been established. Moreover, by comparison with traditional methods, the greater efficacy of the proposed procedure has been demonstrated.

To assess the cleaning efficacy of these hydrogels, a multidisciplinary approach, combining non-invasive spectroscopic infrared techniques together with scanning electron microscopy, chromatographic (HPLC) analysis and pH investigations has been used. Near infrared spectroscopy spectra were coupled with a chemometric analysis to achieve a better interpretation of data.

This work constitutes a preliminary step towards focused study in the development of α-cyclodextrin/polymer hydrogel family which will allow cleaning of paper artifacts with peculiar characteristics.

## Background

Paper artifacts are difficult to restore, due to their inherent fragility, their degradation processes and their multi-component composition. Many critical steps, often carried out during the restoration of paper materials, are the cleaning of the sheets (i.e., the removal of the dull patina), the removal of adsorbed heavy metals and of glue, and the optimization of pH as well as of the degree of humidity [1,2]. In particular, the cleaning of paper, usually achieved by means of solvents (organic or not), presents several problems, such as swelling and dissolution of some components during treatment; furthermore, solvents could be harmful for operators [2].

In the last years, to confront these issues, innovative cleaning methodologies have been proposed based on application of suitable hydrogels. Due to the high retention power and viscosity of gels, the penetration of the liquids into the paper sheets is significantly reduced, therefore minimizing damages [2-5]. However, to impair dangerous microbial growth [6,7], a complete removal of the gel is required and such a procedure often requires abrasive mechanical action (i.e., removal with scraper) or solvents, often unsafe for the artwork. In this contest, rheoreversible hydrogels [8] represent a useful alternative to overcome many known problems.

A well-known family of rheoreversible hydrogels consists in complexes between polymers, like polyethylene oxides (PEO) or several pluronic copolymers (like poly(ethylene oxide)_20_-poly(propylene oxide)_70_-poly(ethylene oxide)_20_ (PEO_20_-PPO_70_-PEO_20_, in the following PLU), and α-cyclodextrin (in the following, α-CD) [9-13] whose gelation is promoted by physical cross-links induced by supramolecular self-assembling [9-11]. Hydrogels based on physical cross-links are able to transduce external stimuli (like pressure) into macroscopical changes of their rheological properties (i.e. swelling) [11,14-18]. Moreover, these hydrogels are thixotropic and reversible [9,10,17]. Therefore, their removal could be easily performed with a gentle use of soft mechanical action, like the use of a soft brush, without involving invasive methods. These hydrogels possess other peculiarities that render them extremely suitable in the paper restoration field. For example, they are safety for the operators as they are already used in medicine because they are biocompatible and non toxic [9,10,12,13]. The hydrogel properties and also the molecules release from them are not sensitive to pH and do not require the presence of specific ions [13] allowing a fine tuning of the conditions adopted (i.e., presence of divalent instead of monovalent, ions, use of alkaline pH [19]). Moreover, hydrogels can be easily loaded with the desirable cleaning agents, like digestive enzymes, allowing the *in situ* encapsulation of molecules by using mild conditions at room temperature [9]) that are essential to the functionality of enzymes. Unlike other gels used for cleaning paper artworks [20], these hydrogels have other advantages: they are made up of synthetic molecules and therefore less susceptible to microbial attacks, and have amphiphilic properties that, as described in more detail below, could be useful to remove hydrophobic contaminants.

In this paper we report the results obtained using two of these rheoreversible hydrogels as cleaning agents for paper materials. In this study, we have first tested the compatibility of these hydrogels using model materials such as new and artificially aged filter papers. Secondly, these hydrogels have been used to clean paper samples belonging to the XVIII century, establishing the effectiveness of the proposed methodology. Moreover, further investigations have been performed to propose a novel process in the paper restoration field, that is, the possibility to remove hydrophobic patina from paper artworks. For this purpose, each hydrogel has been applied to both new and aged filter paper samples soiled with linseed oil, and the efficiency of this method for cleaning sheets has been evaluated.

Due to the complexity of the problem under examination, that is to assess the validity of this methodology, an appropriate experimental approach is necessary. In this contest, many non-invasive spectrophotometric techniques have been employed. One involves vibrational spectroscopy, in both the mid and near infrared region, the results of which are extremely useful to study cellulosic material in a not destructive way, [21]. As opposed to Fourier transform infrared technique in the middle infrared region, (mid-FTIR), near infrared (NIR) spectra are usually too complex to obtain useful information simply by the approach of band assignations; therefore, chemometric analysis has been performed on the NIR spectra to obtain a rationalization of data and a better characterization of the systems under study [22]. To confirm the results obtained and further investigate about various paper samples, invasive techniques such as high performance liquid chromatography (HPLC with UV–vis detector) analysis, scanning electron microscopy (SEM) and pH measurements have also been used.

## Result and discussion

### Compatibility and removability studies

Filter paper has been used as a model sample material to test the compatibility and removability of our cleaning agents and also to set up the cleaning procedures. The suitability of filter paper depends on the following properties: it is not degraded, it has not been subjected to bleaching treatment, and it has a neutral pH.

**Figure 1:**
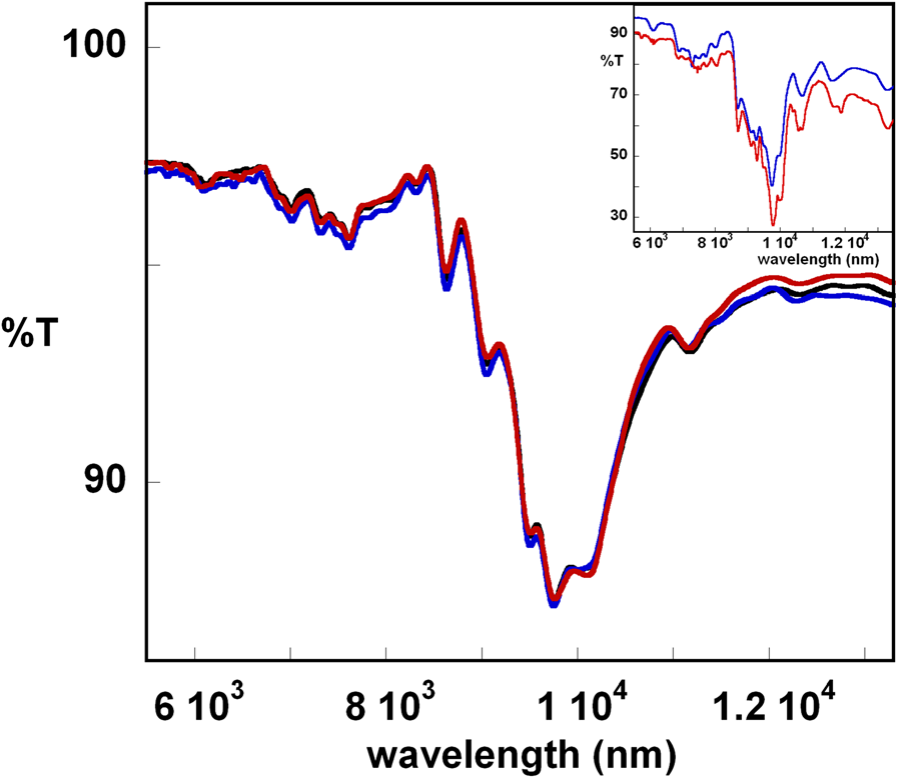
**mid-FTIR ATR spectra of paper.** Paper non treated (red line), paper after PEO hydrogel (black line), or PLU hydrogel (blue line) treatment for 45 minutes: In the inset: mid-FTIR ATR spectra of PEO hydrogel (red line) and PLU hydrogel (blue line) itself*.*

The mid-FTIR spectra, obtained using the attenuated total reflection (ATR) apparatus, of the paper samples before and after hydrogel (PEO or PLU) treatment for 45 minutes, are reported in Figure 1.

The spectra of all the samples show the features typical of cellulose paper in the region 10500–6700 nm, where there are present absorption bands due mainly to CO and CC stretching, antisymmetric in-phase ring stretching, CCH and OCH bending and stretching modes, as well as COH and HCH bending [23,24]. The strong similarity of the reported spectra indicates that the hydrogels are completely removed after treatment, and it also suggests that no detectable chemical degradation of cellulose takes place as a result of the hydrogel treatment, as discussed in more detail below.

To confirm these results, HPLC experiments on water extracts of treated paper samples have been performed. The chromatograms do not show the characteristic peaks that can be attributed to the hydrogels used, thus confirming the absence of hydrogel residues on the paper samples after gel removal (data not shown).

In order to further evaluate the removability and the compatibility of the hydrogels relative to paper treatment, NIR spectra have been recorded from paper samples, either untreated controls or samples after treatment with hydrogels for different times [21,25,26] (see Additional file 1: Figure S1). The spectral data were then analyzed by means of a global PCA [27,28] model which was computed including samples contaminated with linseed oil, as reported in “*Oil removal from fresh and artificially aged samples*” section.

**Figure 2:**
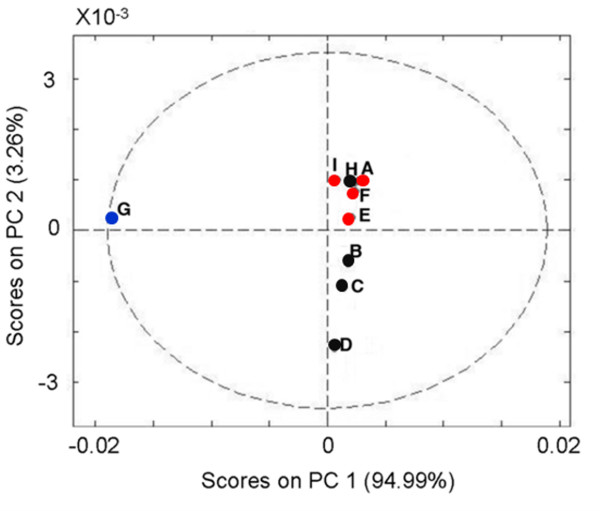
**PCA analysis.** Projection of samples onto the space spanned by the first two principal components (PCs): (A) filter paper; (B-D) filter paper treated with PEO hydrogel for 15, 30 and 45 minutes; (E-F) filter paper treated with PLU hydrogel for 15 and 45 minutes; (G) filter paper loaded with linseed oil; (H) filter paper loaded with linseed oil and treated with PEO hydrogel; (I) filter paper loaded with linseed oil and treated with PLU hydrogel.

The results of PCA analysis on these samples are reported in Figure 2 (sample A-F), in the form of the projection of the analyzed samples onto the space spanned by the first two principal components (explaining more than 98% of the original variance). Inspection of this plot has evidenced that treatment with hydrogels seems to have little effect on the NIR spectrum of paper samples, given that the first principal component (PC1) (accounting almost the 95% of the variability) was little affected by hydrogel treatment (point B-F compared with point A). All the differences between samples involve the second principal component (PC2) which, however, accounts for only a small portion of the spectral variability. These data indicate that the samples before and after hydrogel treatment have similar spectral features, there being no significant differences detected.

The pH values of untreated paper versus that treated with PEO and PLU hydrogels were 7.7, 7.8 and 7.5, respectively, confirming that the hydrogel treatment does not significantly damage paper samples.

### Application on real samples: papers from XVIII century

These hydrogels have been used on a “real sample” (RS) that is a fragment of the book “*Theatrum Veritatis and Justitiae*” (Venezia, 1735), whose main component is cellulose as evidenced by Graff C staining experiments (data not shown) [29].

**Figure 3:**
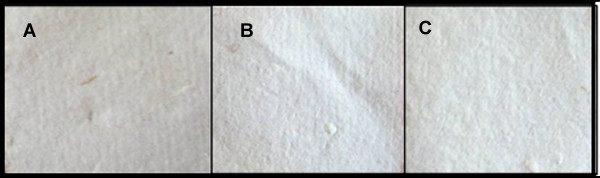
**Cleaning ability of hydrogel.** Direct visualization of **(A)** RS untreated sample; **(B)**: RS sample treated with PEO hydrogel, and **(C)**: RS sample treated with PLU hydrogel.

In Figure 3 a direct visualization of the cleaning ability of the hydrogel is presented. As can be seen, treated papers appear cleaner and brighter than untreated one, indicating that these hydrogels are able to remove the patinas and oxidation products that are responsible for their brownish color [5,26,30].

**Figure 4:**
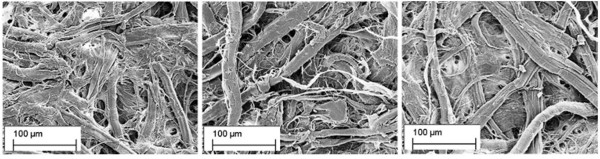
**Study of the hydrogel effects on paper.** SEM images with 100 μm scale bar of RS paper samples (from left to right) untreated, cleaned with PEO, and cleaned with PLU, respectively.

SEM experiments, performed on RS samples, then provided more insight as to the hydrogel effects on papers by visualization at higher resolution. As shown in Figure 4, the hydrogels are able to clean paper samples while no visible degradative processes are provoked [20,31,32].

**Figure 5:**
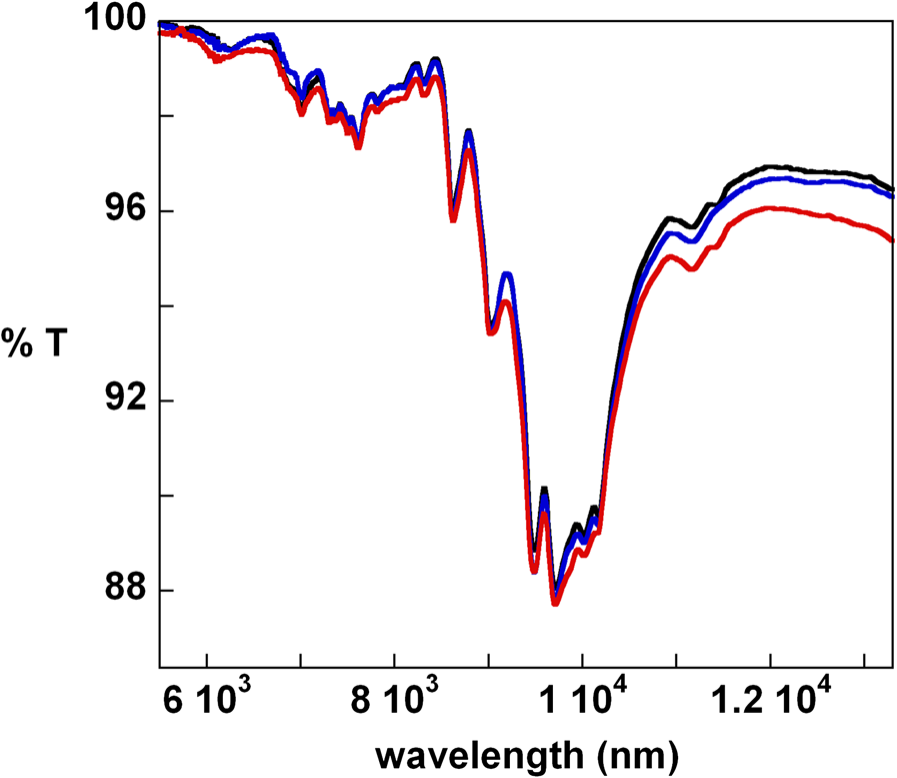
**mid-FTIR ATR spectra of RS samples.** Red line: no treated paper; blue line: paper cleaned using PLU hydrogel; red line: paper cleaned using PEO hydrogel.

Mid-FTIR spectra, of RS samples before and after cleaning with hydrogels are reported in Figure 5. Also in this case, the absence of peaks due to hydrogel residues and the spectral similarity between the samples before and after cleaning corroborate both the compatibility of the hydrogels with cellulosic paper and the efficiency of removal procedure.

**Figure 6:**
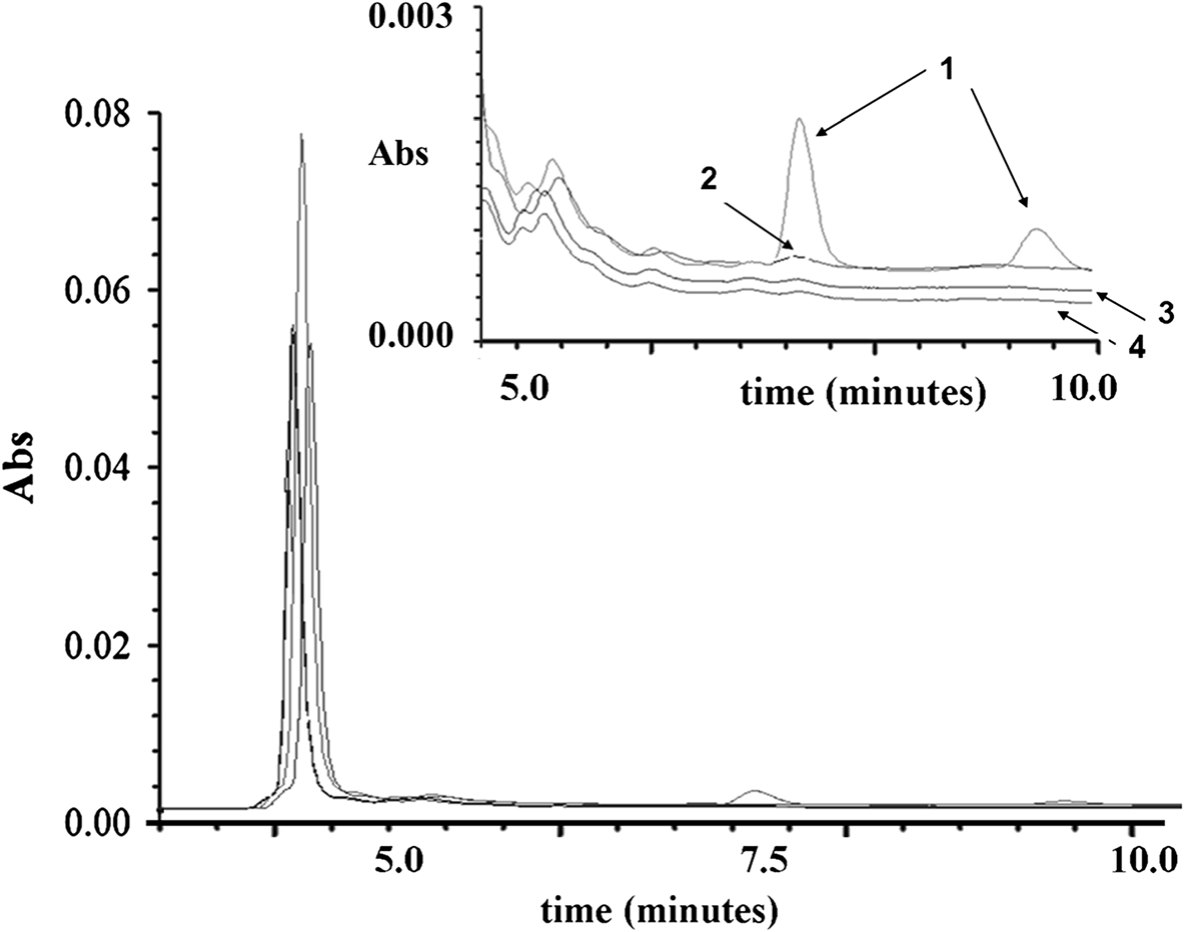
**HPLC analysis.** HPLC chromatograms of the extracts in water of RS before and after cleaning treatment with traditional and hydrogel proposed methods. In insert: detail of chromatogram, region between 4 and 10 minutes before cleaning treatment (1), and after treatment with water (2), with PLU hydrogel (3) or with PEO hydrogel (4). STRATA-SAX separation and concentration of the acid components of each analyzed sample (before and after cleaning treatment) were performed before the chromatographic analysis.

To confirm the safety of the proposed procedures as well as the cleaning capability of the hydrogels, we have performed pH and HPLC measurements before and after treatment, following the same procedures used for studies of filter paper. A comparison with a well-established and traditional method that consists in a treatment with water solution [29], is also reported. The pH measurements confirm the efficacy of all the cleaning methods adopted on RS fragments relative to obtaining optimal pH status. In all cases the pH after treatment increased from pH = 7.8 to the optimal values for cellulosic material, that is from pH 8 to 9. In particular, the pH obtained after PEO hydrogel, PLU hydrogel, and water bath treatments were found to be 9.1, 8.6 and 8.9 respectively) [1]. The removal of acid components has been confirmed by chromatographic analysis performed on the same aqueous extracts, after STRATA-SAX treatment (Figure 6). The acidic components are completely removed by cleaning treatment with hydrogels, as can be deduced from the attenuation and/or disappearance of the peaks roughly centered at 7.5 and 9.5 minutes in the chromatograms obtained from extracts of RS treated with both hydrogels (Figure 6). On the basis of comparison with organic acid standards (data not shown), the peak at 7.5 min is related to malic acid, while the second is probably due to a degradation product of gelatin [33-35]. By contrast, in the case of water treatment only a not complete removal of malic acid is obtained. From this point of view, the hydrogel methods prove to be more efficient than the traditional water washing.

### Cleaning of oil: application on paper

#### Oil removal from fresh and artificially aged samples

**Figure 7:**
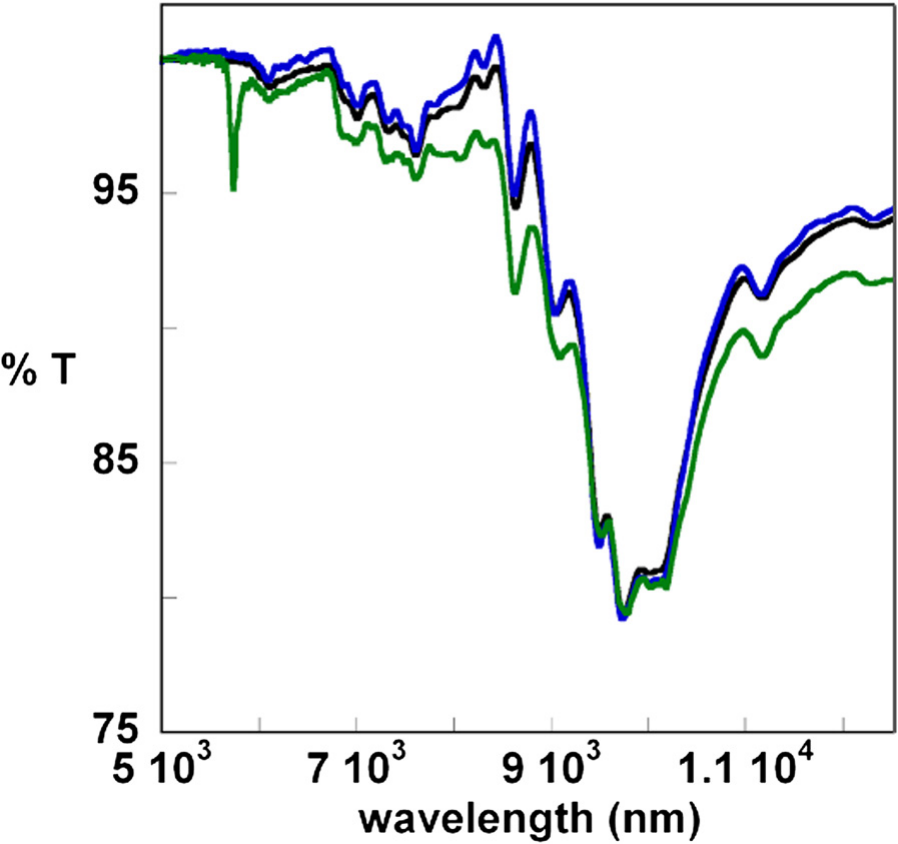
**mid-FTIR ATR spectra of papers containing linseed oil.** The figure shows the results obtained for paper samples before (green line), and after PEO hydrogel (black line), or PLU hydrogel (blue line) 45 minutes treatment.

Water washing is usually unable to clean hydrophobic contaminants from paper samples. These hydrogels, on the other hand, because of their amphiphilic properties, could overcome this pivotal problem without the use organic solvents which are known to be dangerous for paper samples, as well as being potentially harmful for the operator. To mimic the presence of hydrophobic contaminants on paper for this study, filter paper strips have first been impregnated with linseed oil. Linseed oil was chosen because it is a natural substance widely used as a carrier for pigments in inks and as a primer for glossy paper [36,37]; therefore millponds due to this substance are widespread. In the first phase of the investigation, these soiled strips were cleaned using each hydrogel (*fresh samples*). The samples were analyzed by mid-FTIR and NIR spectroscopies before and after the cleaning treatment. The comparison of the FTIR spectra obtained from these fresh paper samples (soiled with linseed oil, before and after the cleaning step with PEO or PLU) is reported in Figure 7. In this figure, it is possible to observe that after gel treatment, absorption peaks due to oil (the main one is localized at 5747 nm and is relative to carbonyl stretching) in the spectra disappear, therefore indicating that oil is totally removed by use of the hydrogels. In addition, NIR spectroscopic analysis on the same samples has been performed (see Additional file 1: Figure S1) confirming the mid-FTIR ATR results.

As reported earlier, as a means to rationalize NIR data through chemometric analysis, PCA has been carried out. In Figure 2, PCA results are reported (points G-I). This analysis is clearly able to reveal the sample containing linseed oil (point G), and consequently the ability of these hydrogels to remove it. In particular, paper samples soiled with oil and then treated with PLU or PEO hydrogels for oil removal (H, I respectively) fall in a region of the PC plot very close to the untreated paper samples (A). As regards the interpretation of the observed differences among paper samples, those treated with oil (sample G) versus the clean and hydrogel-treated papers (all points different from G), inspection of the loadings for the first principal component suggests the spectral region between 2222 and 2353 nm as highly contributing and, to a lesser extent the regions between 2020 and 2105 nm as well as those between 1883 and 1925 nm. Indeed, in these regions, bands attributable not only to paper samples, but also to oil, are present [21,38].

In order to evaluate the possibility of long term effects of the application of the investigated hydrogels on paper, the same “fresh” samples, analyzed by IR spectroscopy and discussed above (Figures 2, 7 and Additional file 1: Figure S1), were exposed to the artificial aging protocol reported in Experimental section. NIR spectra were subsequently registered (see Additional file 1: Figure S2). Finally, the two sets of spectra (before and after aging) were gathered in a single matrix and analyzed by a second PCA procedure following 1st derivative correction and mean centering.

**Figure 8:**
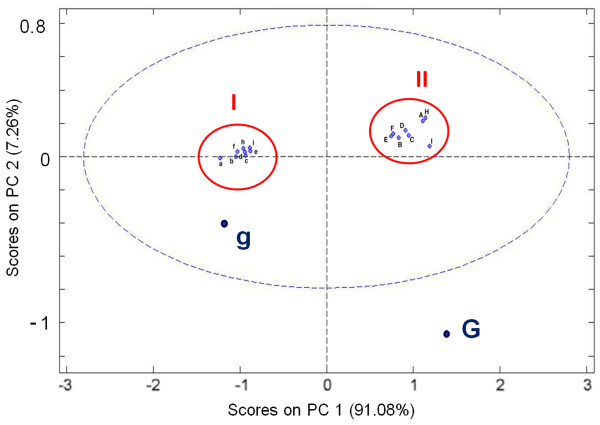
**PCA analysis.** Projection of samples onto the space spanned by the first two principal components (PCs). (lowercase and uppercase letters are related to the aged and non aged samples); (A,a) filter paper; (B-D, b-d) filter paper treated with PEO hydrogel for 15, 30 and 45 minutes; (E-F, e-f) filter paper treated with PLU hydrogel for 15 and 45 minutes; (G,g) filter paper loaded with linseed oil; (H,h) filter paper loaded with linseed oil and treated with PEO hydrogel; (I,i) filter paper loaded with linseed oil and treated with PLU hydrogel. Red circles are for clarity.

The projection of the samples onto the first two principal components (accounting for more than 98% of original variance) is reported in Figure 8. In this figure, it is possible to observe a clear distinction between “aged” (group I and sample g) and “fresh” samples (group II and G), which are separated along the first principal component. On the other hand, along the second principal component the effect of cleaning can be seen, as there is a clear separation between samples G and g, containing oil, and the cleaned ones (groups I and II). Moreover, it can be seen that no significant influence of hydrogel treatment in “aged” paper samples can be observed, as all the points assigned to “aged” samples fall in the same region of the PC plot (except the one corresponding to the sample on which oil was applied but not removed).

As in the previous case, interpretation of the observed differences among samples in terms of original spectral variables can be performed by inspection of the loading plot (data not shown). In particular, when considering the second principal component (that mainly accounts for the differences between samples on which linseed oil was applied and not removed, and the others), spectral regions appearing to contribute the most to the definition of the PC are very similar to those (1^st^ component in Figure 2) identified in the case of not aged samples: (2236–2359 nm and 1868–1908 nm). On the other hand, as far as the first principal component (accounting mainly for ageing effect) is concerned, the variables which show the highest absolute values of the loadings are the intervals 1394–1434 nm, 1865–1925 nm and 2015–2101 nm [21,38,39]. The chemometric processing thus shows that NIR spectroscopic analysis can be a useful tool to discriminate between different contaminants of papers, being able to reveal surfaces contaminated with oil. Furthermore it can provide information relative to the effects of ageing on paper artworks.

#### Hydrogel application on artificially aged samples

**Figure 9:**
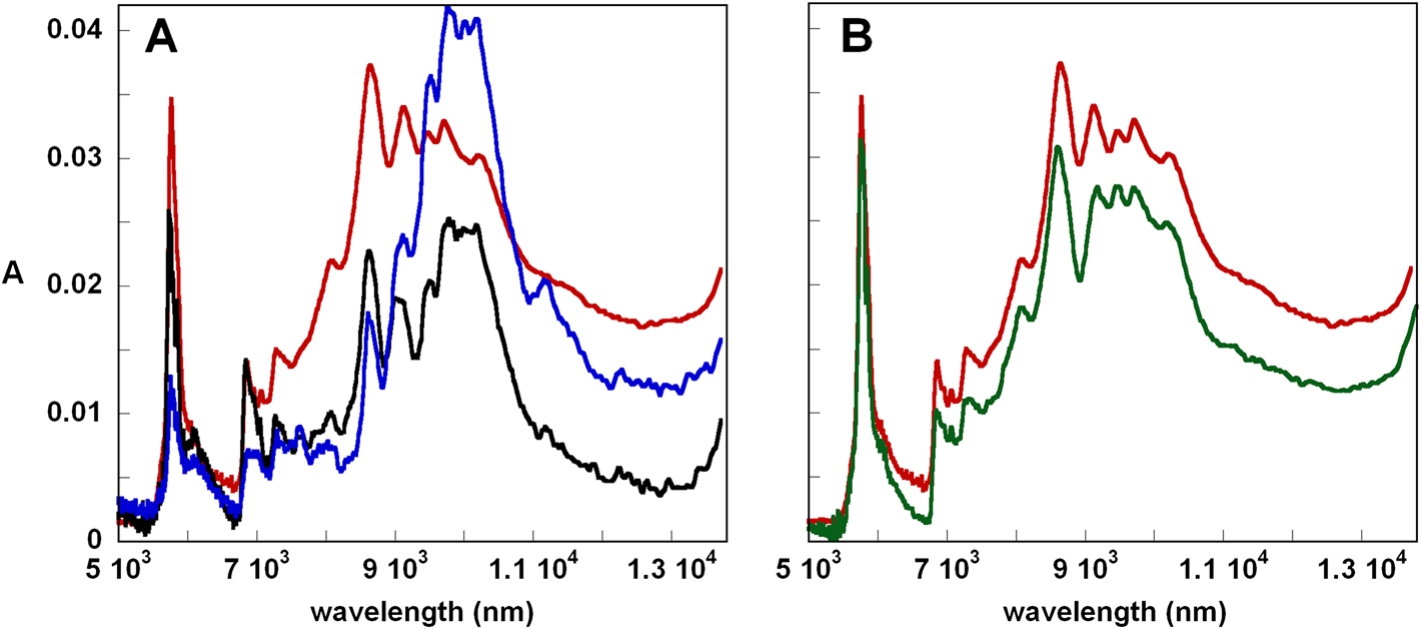
**mid-FTIR ATR spectra of soiled paper samples, aged and cleaned.** The figure shows in panel **A** the results obtained for paper samples untreated (red line), treated with PEO hydrogel (black line), or with PLU hydrogel (blue line); and in panel **B** untreated (red line) and washed with water (green line).

The experiments discussed above (as to the effect of aging of the samples following hydrogel treatment) provide evidences that these cleaning procedures do not promote premature aging of treated papers. However, the majority of paper samples eventually subjected to restoration are already aged. To reproduce these conditions we have artificially aged the filter paper strips impregnated with linseed oil and then used our hydrogels to clean these “aged” samples. The efficiency of hydrogel cleaning under these circumstances was then compared to that of a standard cleaning method, namely washing with water. Analysis by mid-FTIR ATR spectroscopy is able to show the two hydrogels, and particularly PLU, have partially removed the oil, while the water treatment was not able to do so (Figure 9). Indeed, in this respect, the absorbance ratio between two peaks, one at 5747 nm due to oil, and the other at about 10111 nm mainly due to cellulose, seems to be particularly diagnostic. This ratio changes only slightly, going from 1.16 for the untreated sample to 1.12 for paper cleaned with standard method. By contrast, the ratio is lowered to 1.02 for a sample treated with PEO, and falls sharply to 0.32 in the case of the sample cleaned with the PLU hydrogel. These data indicate PLU as a far more efficient agent for oil removal.

**Figure 10:**
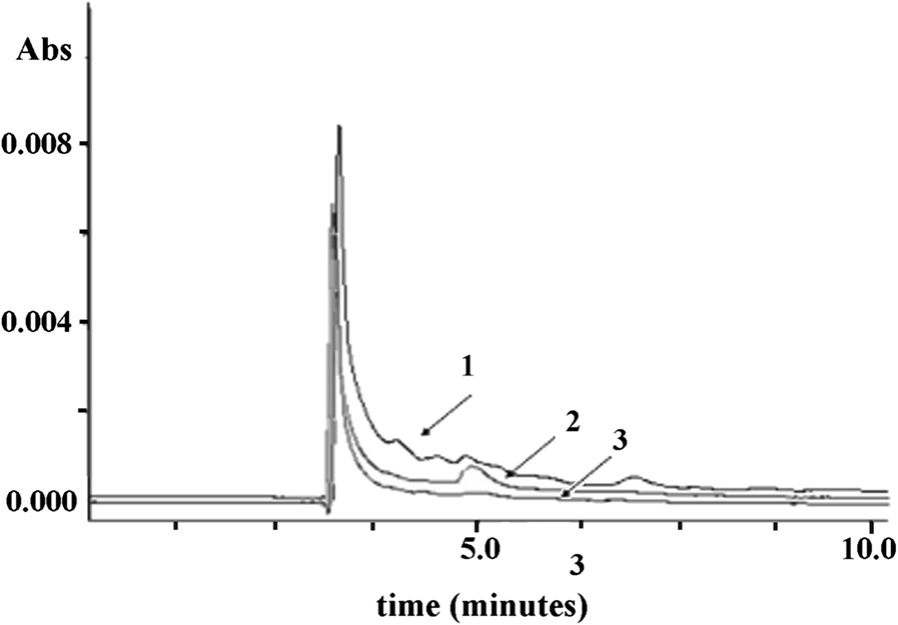
**HPLC analysis for soiled filter paper, soiled and cleaned.** The figure shows the results obtained for the filter paper with aged linseed oil before (line 1) and after PEO hydrogel (line 2) and PLU hydrogel (line 3) treatment.

Chromatographic analysis then confirmed the ability of both hydrogels to remove the aged linseed oil from filter paper more effectively than the traditional method based on a water bath. In Figure 10 the cleaning efficiency of both hydrogels as to oil removal are compared. In this case, the chromatograms are less well resolved due to the presence of high molecular weight components present in the aqueous extracts of the samples treated with the three cleaning methods. In Figure 10, the presence of the oil in the chromatograms is evident, particularly for extracts from the filter paper with aged linseed oil, whether treated or not with the PEO hydrogel. Confirming the results obtained with mid-FTIR ATR analysis, better results were obtained when PLU hydrogel was used, as shown in the chromatogram in the region between 3–10 min (line 3). In fact the chromatogram of filter paper with aged linseed oil shows the characteristic degradation products peak of cellulose around 3 min and a region rich in acidic components between 3.5 and 9 min (the results were compared with the chromatograms obtained for water extract of aged pure cellulose and salts of alkaline reserve – data not shown). This region shows markedly attenuated peaks in the chromatogram of the sample cleaned with PLU hydrogel, while a well-defined peak at 5 min remains when the sample was treated with the PEO hydrogel. By contrast, the cleaning treatment with PLU hydrogel has removed all oil residues.

pH measurements have shown that the alkalinity of paper after treatments increases, as the pH changes from 7.1 for the untreated sample, to 7.2 and 7.5 for samples cleaned with PLU and PEO hydrogel respectively, indicating that both hydrogels, and in particular PEO, are able to remove the most acidic components of oils, as already detected in the case of naturally aged paper (sample RS). It should be noted that further studies have to be developed in order to examine the effect of these hydrogels on written paper artworks.

It should be noted that the two hydrogels, while both being effective for cleaning, have slightly different properties. As reported earlier, for instance, the pH values of paper samples treated with PEO hydrogel are higher than that of fragments cleaned with PLU hydrogel, indicating that the latter hydrogel is better able to remove the soluble acid fractions from paper. At the same time, the PLU hydrogel more effectively removed hydrophobic materials like oils from samples than did PEO hydrogel. These results can be explained by taking into account the differences in hydrophobicity of the two polymers. PEO is constituted by ethylene oxide residues and therefore is more hydrophilic than PLU whose residues are ethylene oxide and propylene oxide (in a 2:1 ratio) [40,41]. The difference in hydrophobity is also evident from the difference in retention times of PLU and PEO hydrogels; PEO hydrogel peaks, due to different polar components, have shorter retention times than those of PLU (mean peak: 3.7 min for PLU and 3.2 for PEO, see Additional file 1: Figure S2). As reported in the literature [9], PPO residues in hydrogels formed by PLU polymer and α-CD tend to aggregate among themselves, forming hydrophobic areas within the hydrogel.

These observations are very important in the context of this work, as they illustrate that the alternative use of variations of the proposed hydrogels can give optimal results under different conditions. A restorer, could, in principle, investigate the nature of the patina by use of spectroscopic and chromatographic techniques along with pH measurements, and subsequently decide the kind of hydrogel to be used. The simplicity and similarity of hydrogel preparation procedures is an additional advantage in this respect; as the only difference between the two hydrogels lies the nature of the polymer. A task for the future is therefore to investigate and develop new hydrogels belonging to this family in order to satisfy diverse needs of restorers.

### Experimental

#### Reagents

α-Cyclodextrin (α-CD) was purchased from Fluka (Fluka Chemie, Buchs, Switzerland). The polymers, PEO (MW = 100000 Da) and PEO_20_-PPO_70_-PEO_20_ (PLURONIC P123, MW ~ 5800 Da), were Aldrich products. Solvents such as methanol were of spectroscopic and chromatographic grade and were from Carlo Erba Reagenti (Carlo Erba Reagenti srl, Milano, Italy). All reagents used were of analytical grade and used without further purification.

Real paper samples (RS), are paper sheets part of the printed volume “*Theatrum Veritatis and Justitiae*” Venezia, 1735.

#### Hydrogel preparation

We followed, with slight modifications, the hydrogel preparation general protocols reported elsewhere [9,10,42]. To prepare the hydrogel based on α-CD and PEO (called PEO hydrogel), an aqueous solution of α-CD (0.372 g/ml) was added to an aqueous solution of PEO (0.134 g/ml), while to prepare the hydrogel based on α-CD and PEO_20_-PPO_70_-PEO_20_ (PLU hydrogel), the initial α-CD and PLU hydrogel concentrations were 0.372 g/ml and 0.556 g/ml, respectively. In both cases, cavitand and polymer solutions were mixed in a 1:1 volume ratio, vortexed for several minutes and then gently stirred for almost an hour, at room temperature. Only the PLU hydrogel was subsequently kept at 4°C overnight. Hydrogels were stable at 4°C for several weeks.

#### Hydrogel application procedure

For all paper samples under examination, the hydrogel application procedure follows the following protocols.

For compatibility and removability studies, each hydrogel was applied on filter paper samples, with a spatula and left to act for a fixed time (15, 30 or 45 minutes); then, it was removed with a soft brush.

Subsequently, to test the long term effects of hydrogel treatment, the cleaned papers were artificially aged in an oven at 90°C (RH = 25%) for 10 days [43].

For investigation of a real sample, the cleaning procedure was carried out by applying each hydrogel for 45 minutes on fragments (RS) from a printed volume “*Theatrum Veritatis and Justitiae*” Venezia, 1735, and then removing them with a soft brush.

To evaluate the efficacy for removing hydrophobic patina, each hydrogel was applied for 45 minutes on various filter paper samples that had been soiled with linseed oil. In some cases, artificial ageing for 20 days at 80°C was carried out before starting the cleaning so as to give a sample mimicking actual aged paper samples *(aged)*[44]. However, some samples were treated with hydrogels immediately after their preparation *(fresh).* In all cases, the hydrogels were removed with a soft brush.

To test the long term effects of hydrogel treatment, also in this case, the *freshly* cleaned papers were artificially aged as reported earlier [44]. The results obtained with our procedure on RS samples and with aged soiled filter paper were then compared with those obtained by a “standard” cleaning procedure that uses a water bath for 45 minutes [29].

Before and after hydrogel treatments, all the paper samples were analyzed by using several techniques, as described in the following section.

### Paper sample characterization

#### Spectroscopic analysis

Mid-FTIR spectra were acquired on a Thermo-Nicolet (mod. Nexus 670) instrument (Thermo Scientific Inc., Madison WI), equipped with an attenuated total reflectance (ATR) ZnSe cell for measurement in the 2500–14285 nm region, at a nominal resolution of 1.5 nm. Spectra were collected by placing the paper samples directly on the ATR cell. A total of 256 scans were collected for each sample.

NIR analysis was performed in a reflectance mode using a Thermo Nicolet 6700 spectrometer (Thermo Scientific Inc., Madison WI) equipped with an integrating sphere module, a halogen-tungsten source and an InGaAs detector. NIR spectra were collected by placing the paper samples directly on the hole of the sphere. For each sample, 82 scans in the range 1000–2500 nm at a 1.32 nm nominal resolution were acquired.

The microstructural features of paper samples were investigated by use of a field emission scanning electron microscope (FE-SEM) Leo Supra 35 (Oberkochen, Germany) under ultra-vacuum at an accelerating voltage of 10 kV. Before performing experiments, the samples were coated with Au to enhance sample conductivity.

#### Chemometric analysis

The results of NIR analysis were processed by means of a chemometric exploratory data analysis technique, namely Principal Component Analysis (PCA) [27,28]. The aim of PCA is to compress the data set by projecting the samples on a low-dimensional subspace without losing the relevant information. The way the algorithm achieves this goal is by defining the axes of this subspace (called Principal Components) as those along which the variance of the projected data is maximized, under the additional constraint of orthogonality. Mathematically, this concept takes the form of the bi-linear model:

$$X=TP^{T}$$

where **X** is the matrix of the original experimental data, **T** is the matrix containing the coordinates of the samples in the space of the principal components (*scores matrix*) and **P** is a matrix describing the contribution of the original experimental variables to the definition of the principal component space (*loadings matrix*). First derivative (computed using a Savitzky Golay approach with a 15 data point window and a 3^rd^ degree interpolating polynomial) and mean centering were used as spectral pretreatments prior to computation of the PCA model [45]. Chemometric analysis was carried out in the Malab (The Mathworks, Natick, MA, USA) environment using routines written in-house.

#### Chromatographic analysis and pH measurements

HPLC analyses were performed with a THERMOQUEST instrument (Shimadzu, Kyoto, Japan), equipped with two pumps and an UV/Vis detector LCGA SPD-10A (Shimadzu, Kyoto, Japan). The apparatus is equipped with a controller SN 4000 (Shimadzu, Kyoto, Japan) that can process data in real time through the CHROMQUEST software. The chromatographic analysis was performed on extracts obtained by treating 1 cm^2^ of every sample (paper or hydrogel) with 1 mL of distilled water, stirring overnight at room temperature. The composition of the mobile phase was 25 mM phosphate buffer of aqueous solution at pH 2.4 and 1% (v/v) methanol. The chromatographic column used was a C18 column (5 μm 150 × 4.6 mm ID - VYDACTM, WR Grace & Co, USA) with a flow rate of 0.7 mL/min, a loop of 20 μL and using a detection wavelength equal to λ= 210 nm [46]. The analysis was performed before and after a cleaning treatment with hydrogel and/or a traditional method (water bath [29,47]). Each chromatographic analysis was repeated three times in the same day (reproducibility intra-day) and on different days (reproducibility inter-day) for all the samples (filter paper, RS samples, filter paper with aged linseed oil).

Only for the analysis of filter paper and RS, an anion exchange column (STRATA-SAX Phenomenex, Torrance, CA, USA) was used for the separation and concentration of the acid component of each analyzed sample [48]. HPLC has been coupled with preliminary purification by solid phase extraction (SPE) cartridge on the water extracts of the paper samples. The SPE method was used for separating, for concentrating and for converting all salts present in the paper samples (as alkaline reserve or paper degradation) in the corresponding acid forms, which are easier to be identified by HPLC. In particular the attention was focused on ascorbic, malic, lactic, oxalic, citric, and succinic acids.

Measurements of pH were carried out on the aqueous extract, obtained as previously described, before and after the water and/or hydrogel treatments [45,49] by using an Amel Instrument 334-B pHmeter with a combined glass electrode Ag/AgCl 6 mm (Amel Instrument, Italy); RSD is 5% calculated on three measurements of the same sample.

## Conclusions

In this study, the efficacy of innovative cleaning agents for paper artworks has been assessed. The proposed systems are based on rheoreversible hydrogels, made of α-cyclodextrin and amphiphilic polymers (polyethylene oxide or pluronic). Their features make possible a total and easy removal simply by means of a soft brush, thus avoiding damage to paper artwork. Furthermore, these hydrogels are biocompatible and safe for operators.

To carry out this study, the employment of several different techniques was required. Firstly, the use of non-destructive vibrational spectroscopies and of chemometric analysis (applied to NIR data), has provided fundamental information demonstrating the compatibility of the agents with paper as well as the cleaning capability of these hydrogels. HPLC, pH and SEM measurements have then allowed us to characterize the results obtained in more detail and provide further insight into the cleaning properties of the hydrogels.

In summary, by use of this approach, applied to both real samples and to model systems mimicking paper artifacts, it has been possible to successfully clean both new and aged paper materials, without damage and also without activating anomalous long-term degradation. Interestingly, we have shown that, due to the amphipathicity of the polymers used, these hydrogels are able to remove linseed oil from paper, contrary to traditional water pack treatment. This last preliminary result is particularly significant as it represents a novel approach for developing a safe (relative to both paper and operator) procedure for the removal of hydrophobic patina or coatings without using organic solvents. To the best of our knowledge, the currently reported procedures to remove hydrophobic patina involve organic solvents or surfactants; these can endanger the integrity of the treated sample and are potentially dangerous for operators.

Furthermore, we have demonstrated that the particular cleaning properties of these hydrogels vary depending on the nature of the polymer used. This means that one can easily obtain the most suitable hydrogel to confront the cleaning of a particular paper sample simply by choosing the appropriate polymer for the hydrogel preparation while still using the same hydrogel preparation protocol and the same application procedures.

From this point of view, our works constitutes a first but fundamental step towards an in depth and focused study devoted to the development of rheoreversible α-CD/polymer hydrogels which will allow cleaning and restoration of paper artifacts with peculiar characteristics. By comparison with traditional methods, the greater efficacy of the proposed techniques has been already been demonstrated. Furthermore, our results suggests that, by coupling NIR spectroscopic investigation with chemometric exploratory data analysis can be a suitable approach for determining the presence of contaminants and for assessing the extent of ageing in the paper samples to be handled.

## Abbreviations

mid-FTIR: Fourier transform infrared spectroscopy in the middle region; ATR: Attenuated total reflectance; NIR: Near infrared spectroscopy; HPLC: High pressure liquid chromatography; SEM: Scanning electron microscopy; PC: Principal component; PCA: Principal component analysis; α-CD: α-cyclodextrin; PEO: Poly(ethylene oxide); PLU: Poly(ethylene oxide)-poly(propylene oxide)-poly(ethylene oxide); RS: Real sample; SPE: Solid phase extraction.

## Competing interests

The authors declare that they have no competing interests.

## Authors’ contributions

Claudia Mazzuca and Laura Micheli conceived the study, designed the experiments, carried out acquisition of mid-FTIR, HPLC, and pH data, performed data analysis and data interpretation. Marta Bevilacqua and Federico Marini contribute to NIR experiments and chemometric analysis; Antonio Palleschi co-ordinated the research, participated in the design of spectroscopic experiments, and contributed to the acquisition of SEM data; moreover, together with Gianfranco Bocchinfuso, contributed to data interpretation and helped draft the manuscript. Giuseppe Palleschi participated in the design of HPLC experiments and helped draft the manuscript. All authors read and approved the final manuscript.

## Supplementary Material

Additional file 1 Reports NIR spectra employed for PCA analyses and HPLC chromatograms of the hydrogels and of not treated filter paper. Click here for file
