# Supplementary material for: Rheoreversible hydrogels in paper restoration processes: a versatile tool
Source: Chem Cent J. 2014 Feb 10;8:10. doi: 10.1186/1752-153X-8-10 (PMC3926867; doi:10.1186/1752-153X-8-10)
Supplement: Additional file 1 — Reports NIR spectra employed for PCA analyses and HPLC chromatograms of the hydrogels and of not treated filter paper. [file 1752-153X-8-10-S1.docx]

**Additional file 1**

**TITLE:**

**Rheoreversible hydrogels in Paper Restoration Processes: a versatile tool.**

Claudia Mazzuca^1^, Laura Micheli^1^, Federico Marini^2^, Marta Bevilacqua^2^, Gianfranco Bocchinfuso^1^, Giuseppe Palleschi^1^, Antonio Palleschi^1§^

*^1^ Dipartimento di Scienze e Tecnologie Chimiche, Università di Roma “Tor Vergata”, Via della Ricerca Scientifica snc, 00133, Rome, Italy.*

^2^ *Dipartimento di Chimica, Università di Roma “Sapienza” P.le Aldo Moro 2, 00185, Rome, Italy.*

NIR Spectra

NIR spectroscopic analysis on samples of paper contaminated with linseed oil, treated and not with PEO or PLU hydrogels have been performed (Figure A1 and A2). An inspection, at a glance, of the spectra confirms mid-FTIR-ATR results, showing that oil was totally removed using PEO or PLU hydrogels, as evidenced by the difference between the spectra before and after treatments.


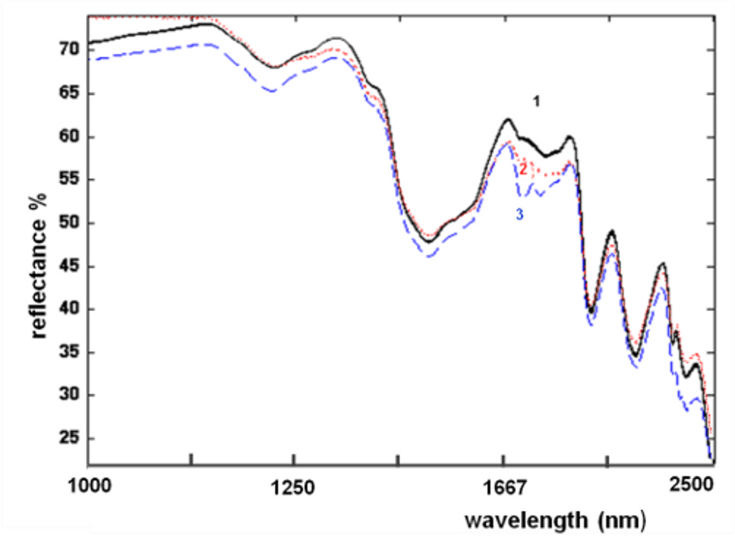


**Figure S1. NIR spectra of several samples:** Group 1: filter paper treated with PEO hydrogel removed after fixed times or paper loaded with linseed oil and treated with PEO hydrogel for fixed times (B-D and H samples in Figure 2 in the article); group 2: not treated filter paper, filter paper treated with PLU hydrogel removed after 15 or 45 minutes or paper loaded with linseed oil and treated with PLU hydrogel for (E, F and I samples in Figure 2 in the article); group 3: filter paper loaded with linseed oil (G sample in Figure 2 in the article).


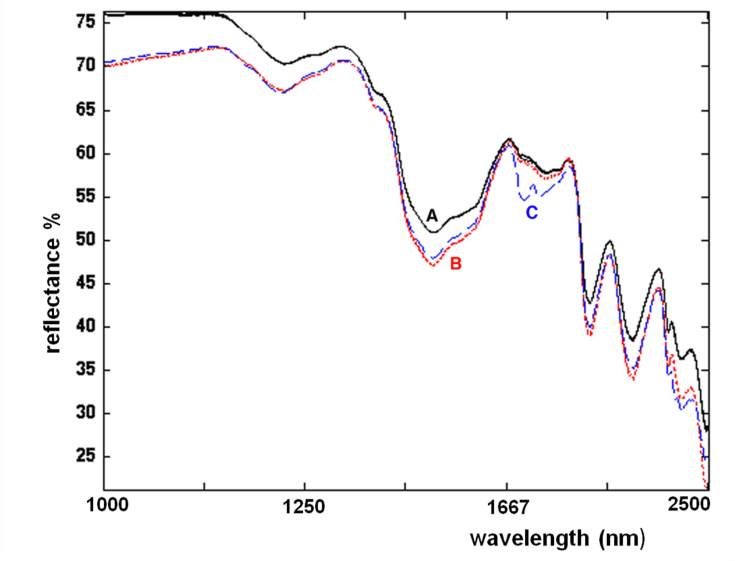


**Figure S2. NIR spectra of aged samples;** Group A: filter papers loaded with oil and then cleaned with PLU or PEO hydrogels (samples h-i, see caption of Fig. 9 in the article); group B: not trated filter paper, filter paper treated with PEO or PLU hydrogel removed after fixed times (samples a-f, see caption of Figure 8 in the article); group C: filter paper treated with oil (sample g; see caption of Figure 8 in the article).

Chromatographic analysis

In Figure A3 the HPLC analysis of PLU gel (line 1), PEO gel (line 2) and fragment of filter paper (line 3) are reported in order to observe the characteristic profile of the gel in comparison to those the paper. The gels are characterized by well-defined and resolved peaks completely absent in the untreated filter paper that shows only an attenuated broadened peak at about 3 minutes associated with cellulose residues. The chromatograms of the gels are characterized by well-defined peaks, particularly at the retention time of 3 minutes, due to some fragment of PLU and PEO gels, characterized by the presence of OC=O groups. In particular, the PEO chromatogram shows other two peaks, due to more complex structure. Also, in this case, the chromatographic analysis was performed on gel portions of 1 cm^2^ and left in water overnight.


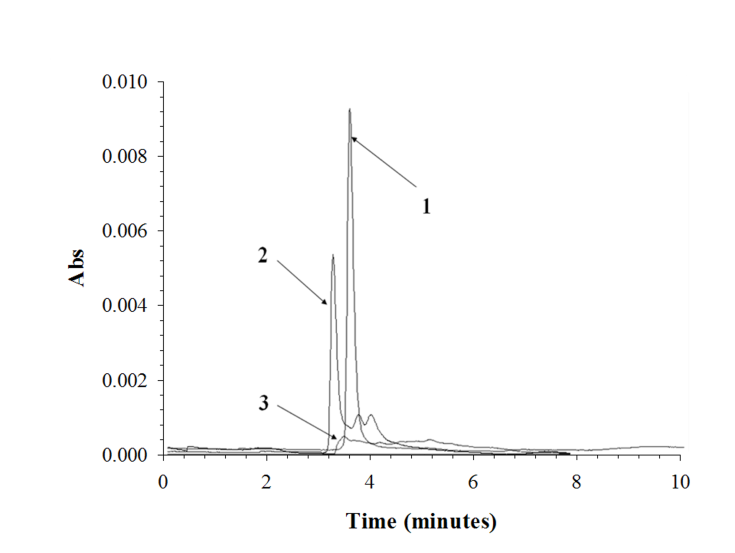


**Figure S3. HPLC analysis of PLU gel (line 1), PEO gel (line 2) and filter paper (line 3).** Samples were extracts in water in order to observe the characteristic profile of the gel in comparison to those the paper.
